# Effects of exogenous salicylic acid on the adaptive capacity of *Leymus chinensis* seedlings under extreme drought conditions

**DOI:** 10.3389/fpls.2025.1720418

**Published:** 2025-12-11

**Authors:** Chongyang Li, Xinying Liu, Bin Li, Rong Yang, Xiangyang Hou

**Affiliations:** 1 College of Grassland Science, Shanxi Agricultural University, Jinzhong, China; 2 Key Laboratory for Model Innovation in Forage Production Efficiency, Ministry of Agriculture and Rural Affairs, Jinzhong, China

**Keywords:** climate change, extreme drought, salicylic acid, *Leymus chinensis*, physiology and biochemistry

## Abstract

To investigate the effects of exogenous salicylic acid (SA) on the growth characteristics of *Leymus chinensis* seedlings under extreme drought stress, this study utilized L. chinensis seedlings as experimental materials and simulated drought conditions using a rainout shelter to exclude natural precipitation. SA solutions at six concentrations (0, 0.25, 0.50, 1.00, 2.00, and 4.00 mmol/L) were applied via foliar spraying. Key growth parameters, photosynthetic traits, chlorophyll fluorescence indices, osmotic adjustment substances, and antioxidant enzyme activities were systematically measured and analyzed. The results showed that exogenous SA significantly alleviated the suppression of seedling height growth caused by extreme drought stress. Under drought conditions, plants treated with 0.50 mmol/L SA exhibited peak levels of chlorophyll (Chl) and proline (Pro) content, both of which were significantly higher than those in the control group (P < 0.05). Additionally, this treatment increased leaf relative water content, soluble sugar (SS) accumulation, superoxide dismutase (SOD) activity, and catalase (CAT) activity compared to the control. Notably, stomatal conductance (gs) was minimized in the 0.50 mmol/L treatment under drought stress, indicating reduced transpirational water loss and improved water-use efficiency. Overall, exogenous SA enhanced the photosynthetic performance, antioxidant defense capacity, and osmotic regulation ability of L. chinensis seedlings. These findings demonstrate that application of 0.50 mmol/L SA effectively mitigates the detrimental effects of extreme drought stress, improves drought tolerance in L. chinensis seedlings, and holds promise for enhancing the resilience and sustainability of grassland ecosystems under increasingly severe drought conditions.

## 1 Introduction

As global warming intensifies, arid regions are experiencing accelerated aridification, a trend that suggests an expanding number of areas will face severe agricultural and ecological drought challenges (Gebrechorkos et al., 2025). According to the Sixth Assessment Report of the Intergovernmental Panel on Climate Change (IPCC), future projections indicate increasing uncertainty in global precipitation patterns (Zhang et al., 2022), along with higher frequency and intensity of extreme drought events (Piao and Wang, 2023). Currently, approximately one-third of the world’s land surface is classified as arid or semi-arid (Zhang x, et al., 2024). Extreme drought—primarily driven by prolonged precipitation deficits—is the most detrimental form of abiotic stress (Bibi et al., 2022), imposing severe constraints on plant productivity (Zhang L, et al., 2024). Drought-induced reductions in crop yields exceed 50% annually (Wu et al., 2023), representing a major threat to global food security. These events not only compromise ecosystem integrity but also result in substantial economic losses (Brás et al., 2021; Farizo et al., 2024). Therefore, investigating the physiological and ecological mechanisms underlying plant responses to extreme drought, as well as developing effective exogenous interventions to improve drought tolerance, is of critical importance to ecological research.

*Leymus chinensis*, a perennial grass species in the genus Leymus of the Poaceae family, exhibits strong adaptive traits including drought tolerance, cold resistance, and salt-alkali tolerance. As a key constructive and dominant species in the eastern Eurasian steppe (Li et al., 2018), it serves as a high-quality forage due to its abundant foliage, high nutritional content, and excellent palatability, making it highly preferred by various livestock throughout the year. Consequently, it has been widely adopted as a superior forage species in pasture systems across many countries (Li et al., 2019). However, drought stress leads to reduced grassland biomass, with particularly pronounced effects observed in the Eurasian steppe region (Bondaruk et al., 2025). Given its dominant ecological role, the growth stability of L. chinensis is essential for maintaining the structural integrity and functional resilience of the grassland ecosystem (He et al., 2021), while its productivity is closely linked to the sustainable development of northern pastoral regions (Shi et al., 2022). Owing to its irreplaceable ecological significance and substantial economic value, L. chinensis is frequently employed as a model species in extensive drought-related studies.

Various strategies have been developed to enhance plant resilience under extreme drought conditions, including improved irrigation and water conservation practices, cultivation of drought-tolerant varieties, optimization of management and planting techniques, and application of exogenous plant growth regulators (Farooq et al., 2024). Among these approaches, foliar spraying of exogenous substances has emerged as a key strategy for mitigating drought stress in plants (Rady et al., 2021). This method offers several advantages: it can rapidly induce drought resistance responses at relatively low cost, enhance antioxidant and osmotic adjustment capacities, and improve physiological and metabolic functions under stress conditions (La et al., 2019). Research has demonstrated that SA reduces cell membrane permeability and enhances antioxidant enzyme activity (Torun et al., 2023), thereby decreasing the accumulation of reactive oxygen species and free radicals, alleviating membrane damage, and mitigating oxidative stress induced by drought (Mohammad et al., 2023). Consequently, SA is widely employed to improve plant tolerance to abiotic stresses such as salinity, drought, and high temperature (Shan et al., 2024).

Although previous studies have demonstrated that SA can effectively mitigate the adverse effects of drought stress on various forage grasses (Safari and Majidi, 2025; Zaid et al., 2019; Khalvandi et al., 2021), the efficacy and underlying mechanisms of SA in *Leymus chinensis* remain poorly understood. In this study, L. chinensis was used as the experimental material, and extreme drought conditions were simulated using artificial rainout shelters instead of the conventional polyethylene glycol (PEG)-based laboratory method (Zhang et al., 2025), thereby more accurately replicating the natural growth environment under drought. Under this novel material–treatment experimental framework, we investigated the effects of different SA concentrations on the growth characteristics of L. chinensis seedlings under extreme drought stress. This approach not only fills a critical knowledge gap regarding SA application in this ecologically important species but also identifies the optimal SA concentration for enhancing drought tolerance in L. chinensis. More importantly, it elucidates the physiological and molecular mechanisms involved in SA-mediated drought resistance, providing practical solutions for grassland vegetation restoration, improvement of grassland productivity, and stable forage supply in arid regions. Furthermore, these findings offer a solid theoretical foundation for the development of drought-resistant cultivation practices and plant-derived anti-drought agents.

## 2 Materials and methods

### 2.1 Experimental materials

The experimental material was Xiwuzhumuqin *Leymus chinensis*, a cultivar previously established by the research team. Seeds were stored at 4°C in a refrigerator and provided by the College of Grassland Science, Shanxi Agricultural University. SA was supplied by Wuhan Proteintech Company.

### 2.2 Overview of the experimental site

The experiment was conducted at the Extreme Drought Experiment Platform, located at the Ecological Grassland and Animal Husbandry Development Research Center of Shanxi Agricultural University in Taigu District, Jinzhong City, Shanxi Province (37.42°N, 112.58°E), at an elevation of 799 m above sea level. The site has a mean annual temperature of 10°C, annual precipitation of 397.1 mm, and approximately 2,527.5 hours of sunshine per year.

### 2.3 Experimental methods

**Figure 1:**
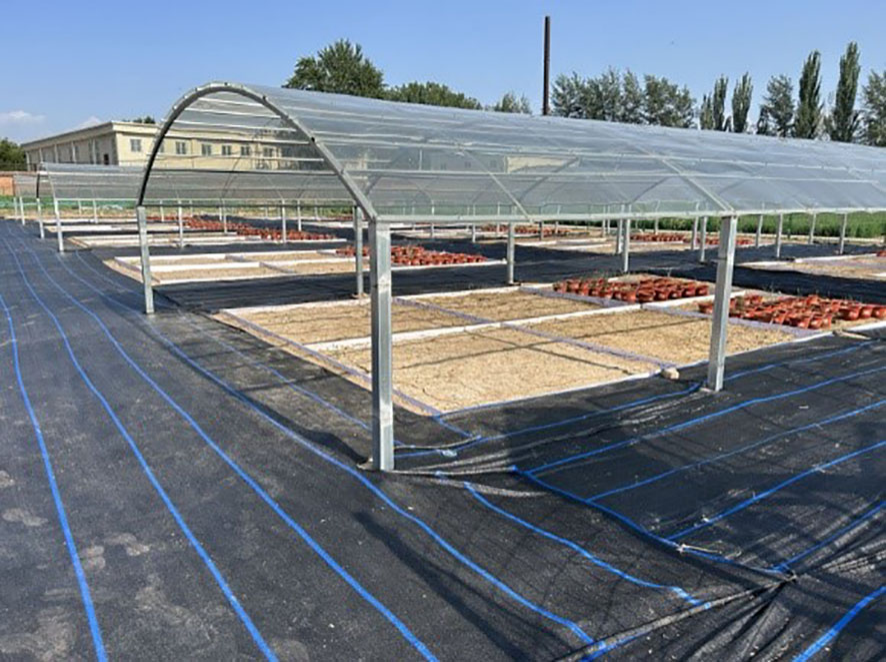
Extreme drought experimental area.

The experimental plots were selected from the “Extreme Drought Experimental Area” located within the drought platform. As illustrated in **Figure 1**, the area was equipped with a roof covered by fully shading yet light-transmitting materials, designed to intercept 100% of precipitation during the growing season while maintaining near-natural light conditions, thereby effectively simulating extreme drought scenarios encountered by plants in natural environments. In this experiment, extreme drought treatment was initiated on July 19, 2024, by withholding all artificial irrigation for potted *Leymus chinensis*. Observations and measurements of relevant physiological and ecological indicators were conducted after 21 days of continuous drought stress. Studies have demonstrated that continuous drought lasting 20 to 25 days can effectively induce significant physiological and biochemical responses in plants (Temel et al., 2017). In this experiment, relevant indicators were measured on day 21 of drought treatment. This time point not only adequately reflects the effects of extreme drought stress but also ensures the survival of the majority of plants, thereby facilitating accurate measurement and systematic evaluation of subsequent physiological parameters.

Uniform-sized, healthy, and plump *Leymus chinensis* seeds were selected. The seeds were disinfected with a 2% sodium hypochlorite solution for 10 minutes and then rinsed 3–4 times with distilled water (Gilbert et al., 2023). Cold stratification was conducted by soaking the seeds in distilled water in a beaker and storing them at 4°C for 20 days to promote germination (Cheng et al., 2022). The treated seeds were sown into 32-cell seedling trays with individual cell dimensions of 53 mm × 27 mm × 58 mm (top diameter × bottom diameter × height). The smooth and flat inner surfaces of the cells facilitated seedling removal during transplantation. The growing medium consisted of a 2:1 mixture of field soil and nutrient soil (Ben Youssef et al., 2025), sterilized by high-temperature treatment [28], with measured nutrient contents of total nitrogen at 1.91 mg/g, total phosphorus at 1.73 g/kg, and total potassium at 18.17 g/kg. After sowing, the trays were transferred to a growth chamber maintained under controlled conditions: temperature at (25 ± 2)°C, photoperiod of 16 h light/8 h dark, and photosynthetic photon flux density of 300 ± 20 μmol m^−2^ s^−1^ (Jiang et al., 2022). Plants were regularly watered until they developed 3–4 true leaves and their root systems formed intact root balls that remained cohesive during handling. At this stage, seedlings were carefully transplanted into cultivation pots to minimize mechanical stress and avoid any physical damage during transfer (Lin et al., 2017). The cultivation pots had dimensions of 28.5 cm × 25 cm × 19 cm (top diameter × bottom diameter × height), and the same growing medium used in the seedling trays was employed. When the seedlings reached a height of approximately 25 cm, the soil was watered to field capacity and watering was then withheld. The pots were transferred to a rainout shelter to impose extreme drought treatment.

Studies have shown that the suitable concentration range of SA varies among different types of gramineous plants due to different planting methods or stress levels (Korgaonker and Bhandari, 2023; Shemi et al., 2021; Bagautdinova et al., 2022), but most of them are concentrated between 0.05 mmol/L and 2.5 mmol/L. As a signaling molecule, the effect of SA may follow the typical “low concentration promotes, high concentration inhibits” effect (Li et al., 2022). To capture the turning point of high concentration during the experiment and make the design more complete, six SA concentrations were set, namely: Control check, 0 mmol/L (CK), Treatment 1, 0.25 mmol/L (T1), Treatment 2, 0.50 mmol/L (T2), Treatment 3, 1.00 mmol/L (T3), Treatment 4, 2.00 mmol/L (T4), and Treatment 5, 4.00 mmol/L (T5).Starting on July 16, 2024, the SA solutions were sprayed onto the leaves each evening until the leaf surface was slightly covered with droplets but without runoff. Approximately 30 mL of solution was applied per pot, and spraying was conducted for three consecutive days. On July 19, drought stress was imposed on *Leymus chinensis*, and observations and measurements were carried out 21 days after the initiation of drought treatment. Each treatment was replicated six times, resulting in a total of 36 experimental units.

### 2.4 Index measurement

#### 2.4.1 Growth index

Plant height: The vertical distance from the base of the *Leymus chinensis* stem to the apex of the uppermost leaf was measured using a graduated tape measure.

Leaf width was determined as the maximum width of the third and fourth uppermost leaves from the apex of *Leymus chinensis*, measured using a graduated tape measure, with the mean value calculated for each plant (Fan et al., 2024).

#### 2.4.2 Physiological and biochemical index

Leaf relative water content (RWC) was measured using the constant-weight oven-drying method according to established protocols (Zhang X, et al., 2025).The fresh weight (mf), turgid weight (mt), and dry weight (md) of leaf tissue were measured sequentially. The RWC was then calculated using the following formula:


$$RWC=\frac{m_{f}-m_{d}}{m_{t}-m_{d}}\times 100\%$$

Leaf chlorophyll content (Chl) was quantified using the ethanol extraction method according to established procedures (Wang et al., 2022), using 95% ethanol as the extraction solvent. Absorbance was measured at wavelengths of 665 nm and 649 nm. The absorbance values were then substituted into the following equations:


$$C_{a}=13.95A_{665}-6.88A_{649}$$

$$C_{b}=24.96A_{665}-7.32A_{665}$$

Chlorophyll a and chlorophyll b concentrations were thereby calculated, and their sum represented the total chlorophyll concentration.

Photosynthetic parameters were measured using a fourth-generation CIRAS-4 portable photosynthesis system. The parameters measured included net photosynthetic rate (Pn), stomatal conductance (gs), transpiration rate (Tr), and water use efficiency (WUE).

The fluorescence parameters were measured using the FMS2+ portable pulse-modulated fluorometer. The indicators measured included: initial fluorescence (Fo), maximum fluorescence (Fm), actual photochemical efficiency (ΦPSII), photochemical quenching coefficient (qP), and non-photochemical quenching coefficient (NPQ).

The contents of soluble sugar (SS), proline (Pro), and malondialdehyde (MDA), as well as the activities of superoxide dismutase (SOD), catalase (CAT), and peroxidase (POD), were determined using assay kits purchased from Beijing Boxi Shenggong Technology Co., Ltd.

### 2.5 Data analysis

All experimental data were processed using Excel 2010 and statistically analyzed with SPSS 26.0 software. One-way ANOVA was performed to assess differences in various indicators across different treatments. Graphs were generated using Origin 2022 software. Principal component analysis (PCA) was subsequently applied to standardize the multivariate data, aiming to reduce dimensionality while retaining as much of the original information as possible. This facilitated a more objective identification of key factors that play a dominant role among the multiple *Leymus chinensis* indicators, and their comprehensive scores were calculated and ranked accordingly. The plspm package was used to construct a partial least squares structural equation model (PLS-SEM) based on exogenous SA concentration and measurements of photosynthetic fluorescence parameters, osmotic adjustment substances, antioxidant enzyme activities, MDA content, and morphological indicators. Path diagrams were visualized using the semPlot package and further refined in PowerPoint 2016.

## 3 Results

### 3.1 Effects of exogenous SA on the morphological characteristics of *Leymus chinensis* under extreme drought

**Figure 2:**
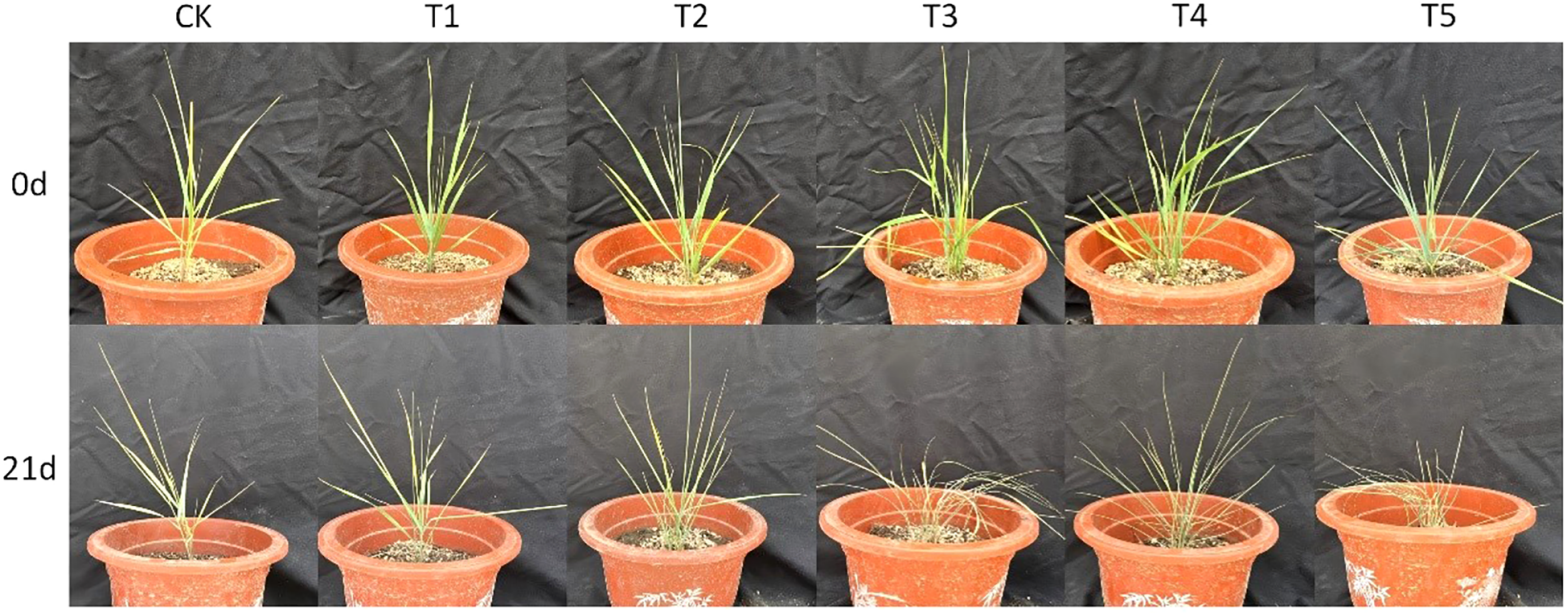
Comparison of plant growth under normal conditions and following extreme drought stress.

**Figure 3:**
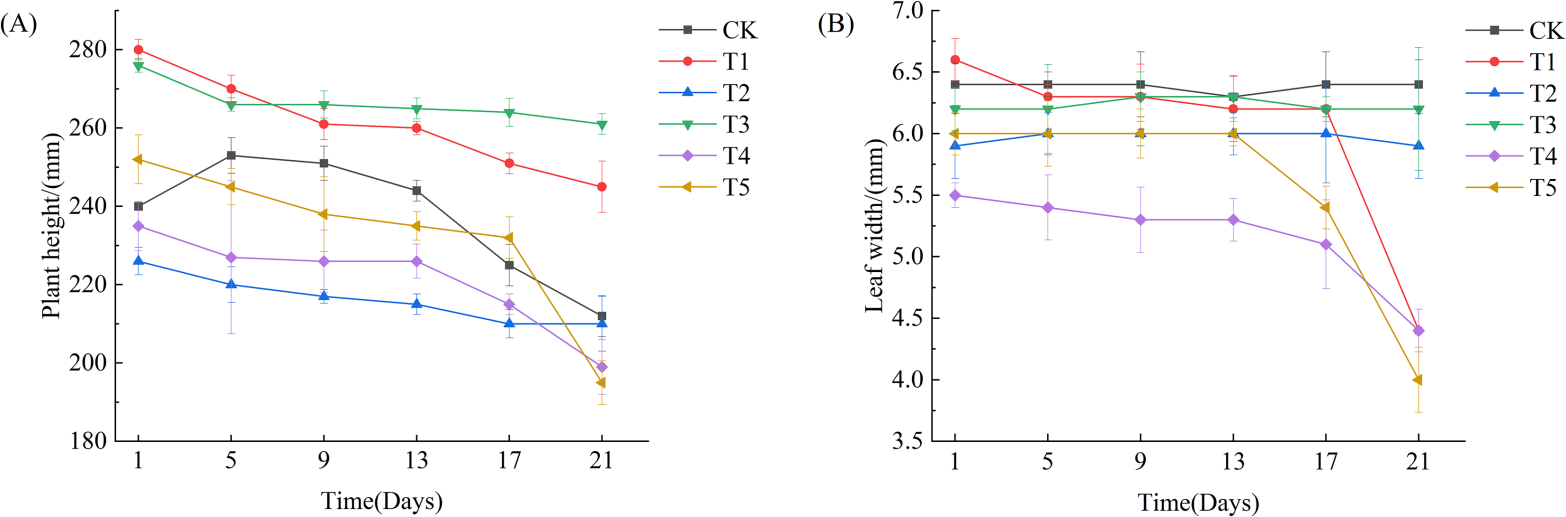
Effects of exogenous salicylic acid (SA) on plant height and leaf width in Leymus chinensis under extreme drought stress. **(A)** Plant height, **(B)** Leaf width. Error bars indicate the standard deviation calculated from six biological replicates.

As shown in **Figures 2** and **3**, after 21 days of drought treatment, the plant height of Leymus chinensis seedlings in all five SA treatment groups decreased with increasing concentration. Notably, the reductions in plant height under T2 and T3 treatments were smaller than that of the control (CK), decreasing by only 0.06% and 0.05%, respectively, relative to CK. From day 13 of the treatment onward, leaf width in the T1, T4, and T5 groups began to decline, whereas no significant changes were observed in the T2 and T3 groups compared to CK throughout the entire stress period.

### 3.2 Effects of exogenous SA on the RWC of *Leymus chinensis* leaves under extreme drought

**Figure 4:**
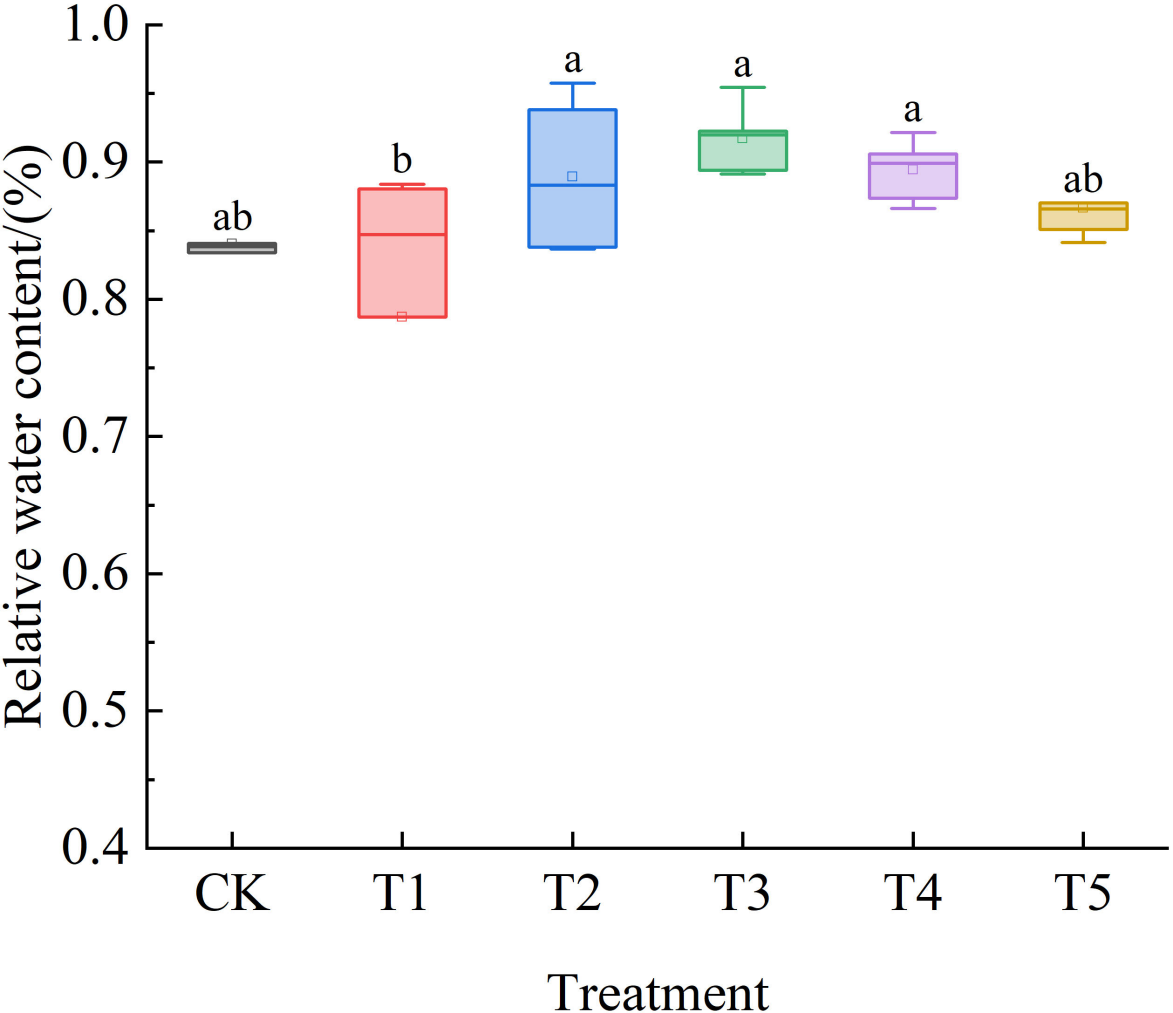
Effects of exogenous salicylic acid (SA) on the relative water content (RWC) of *Leymus chinensis* leaves under extreme drought conditions. Different lowercase letters indicate statistically significant differences at *p* < 0.05.

As shown in **Figure 4**, the RWC of *Leymus chinensis* seedling leaves under the five SA treatments initially increased and subsequently decreased. Compared with the control (CK), the increases were 2%, 5%, 8%, 5%, and 2%, respectively.

### 3.3 Effects of exogenous SA on photosynthetic parameters of *Leymus chinensis* seedlings under extreme drought

**Figure 5:**
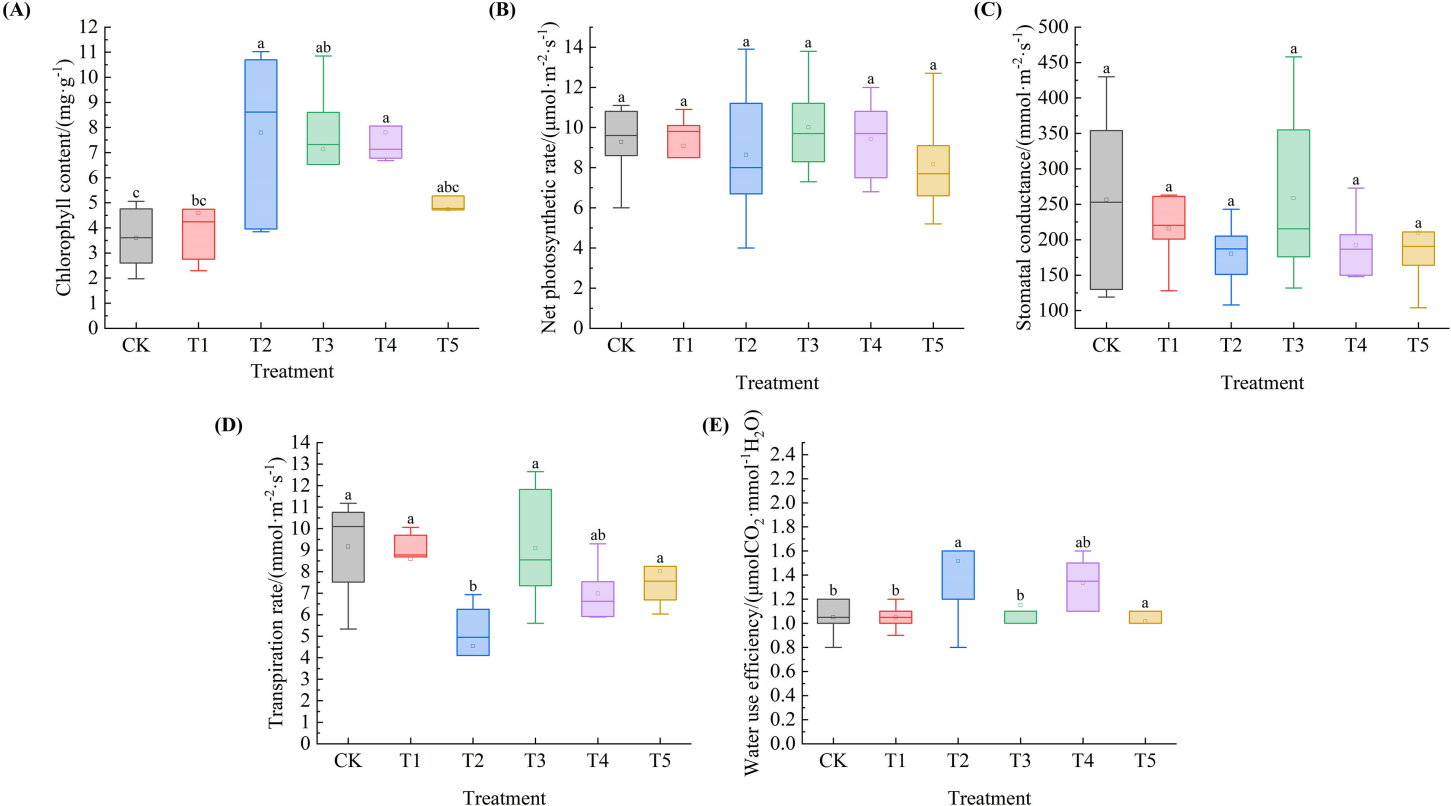
Effects of exogenous salicylic acid (SA) on photosynthetic parameters of *Leymus chinensis* seedlings under extreme drought conditions. **(A)** Chlorophyll content (Chl), **(B)** Net photosynthetic rate (Pn), **(C)** Stomatal conductance (gs), **(D)** Transpiration rate (Tr), **(E)** Water use efficiency (WUE). Different lowercase letters indicate statistically significant differences at *p* < 0.05.

As shown in **Figure 5A**, the Chl of *Leymus chinensis* seedlings exhibited an overall trend of initially increasing and then decreasing with increasing SA solution concentration. Compared with the control (CK), the Chl under the five SA treatments increased by 27.5%, 116.3%, 98.0%, 116.6%, and 31.5%, respectively, with significant increases observed under T2, T3, and T4 treatments (P < 0.05). The Pn of *Leymus chinensis* seedlings in **Figure 5B** exhibited a biphasic decline, characterized by an initial decrease, followed by a transient increase, and subsequent reduction. Relative to the CK, it increased by 7.7% and 1.4% under T3 and T4, respectively. Regarding gs (**Figure 5C**), values under T1, T2, T4, and T5 decreased by 15.9%, 29.8%, 25.2%, and 18.3%, respectively, compared to the control. In **Figure 5D**, the Tr of *Leymus chinensis* seedlings exhibited a fluctuating pattern in response to increasing SA solution concentration, characterized by an initial decline, followed by a transient increase, and a subsequent decrease, and all treatment values were lower than that of the CK, declining by 6.3%, 5.0%, 0.9%, 23.8%, and 12.6%, respectively, with a significant reduction under T2 (P < 0.05). As illustrated in **Figure 5E**, the WUE of *Leymus chinensis* seedlings exhibited a fluctuating pattern in response to increasing SA solution concentration, characterized by an initial increase, followed by a transient decrease, and a subsequent rise. Compared with the CK, it increased significantly by 30.8% under T2 (P < 0.05), but decreased significantly by 2.2% at T5 (P < 0.05).

### 3.4 Effects of exogenous SA on fluorescence parameters of *Leymus chinensis* under extreme drought

**Figure 6:**
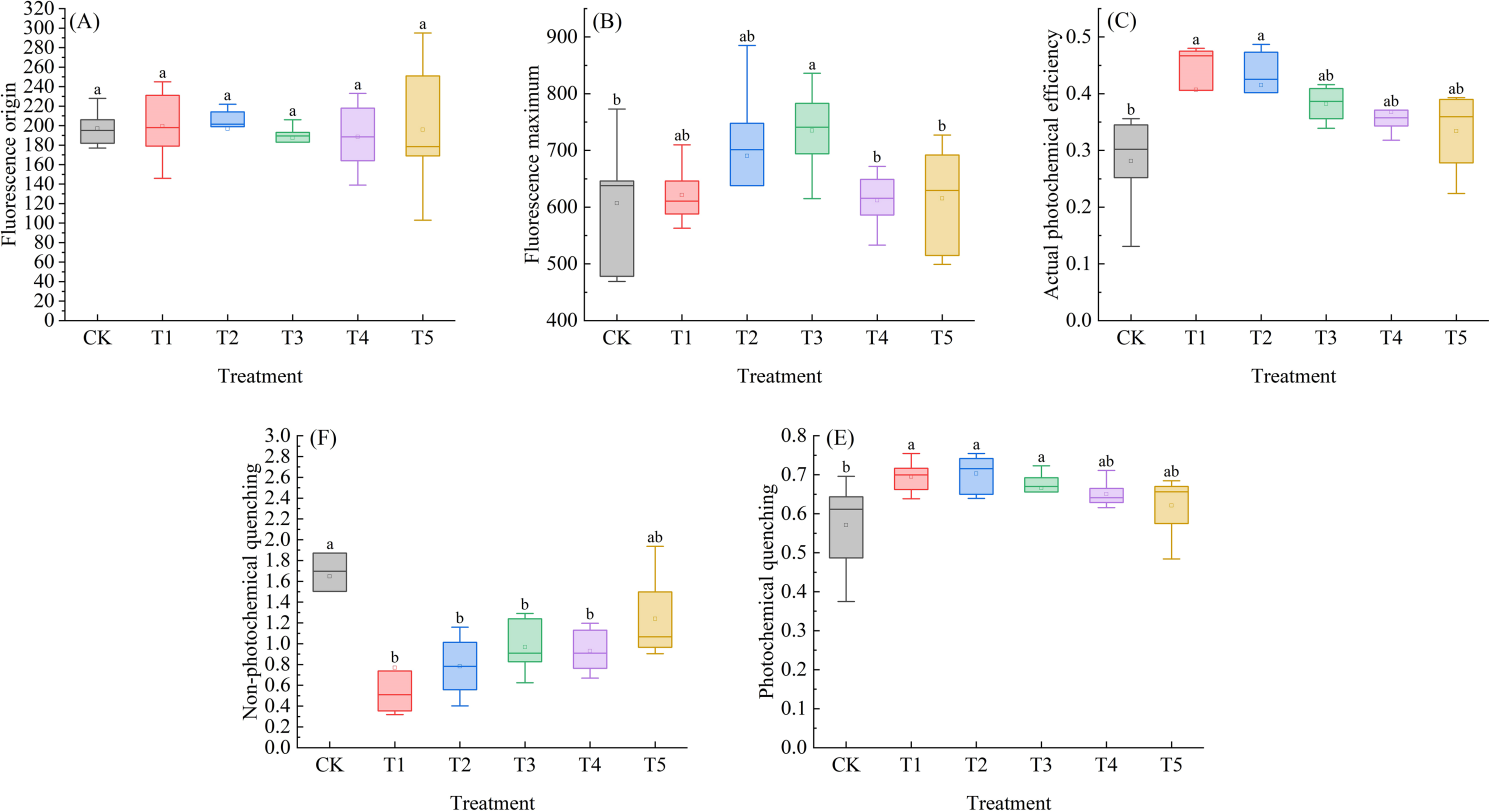
Effects of exogenous salicylic acid (SA) on fluorescence parameters of *Leymus chinensis* seedlings under extreme drought conditions. **(A)** Initial fluorescence (Fo), **(B)** Maximum fluorescence (Fm), **(C)** Actual photochemical efficiency (ΦPSII), **(D)** Non-photochemical quenching coefficient (NPQ), **(E)** Photochemical quenching coefficient (qP). Different lowercase letters indicate statistically significant differences at *p* < 0.05.

As shown in **Figure 6A**, the Fo of *Leymus chinensis* seedlings exhibited a trend of first decreasing and then increasing with rising SA concentration. Compared with the control (CK), Fo under T2, T3, T4, and T5 treatments decreased by 0.2%, 5.1%, 4.4%, and 0.7%, respectively. In **Figure 6B**, the Fm of *Leymus chinensis* seedlings exhibited a dynamic response to increasing SA concentration, characterized by an initial increase, followed by a decrease, and eventual stabilization. Relative to CK, it increased by 2.4%, 13.8%, 21.1%, 0.8%, and 1.4% under the five treatments, with a significant effect observed under T3 (P < 0.05). As shown in **Figure 6C**, the actual photochemical efficiency (ΦPSII) of Leymus chinensis seedlings gradually decreased with increasing SA concentration. Nevertheless, all treatment groups exhibited higher ΦPSII values than the control (CK), with increases of 44.7%, 47.6%, 35.8%, 30.6%, and 18.7%, respectively.; the effects under T1 and T2 were statistically significant (P < 0.05). In **Figures 6D, E**, as SA concentration increased, the NPQ of *Leymus chinensis* seedlings exhibited a gradually increasing trend, yet all values remained lower than those of the control (CK). In contrast, qP progressively decreased with increasing SA concentration, while all corresponding values were higher than those of CK. Specifically, NPQ was significantly reduced by 49.9%, 49.0%, 37.0%, and 39.4% under T1, T2, T3, and T4 treatments, respectively, compared to CK (P < 0.05). Meanwhile, qP was significantly elevated by 21.8%, 23.1%, and 16.7% under T1, T2, and T3 treatments, respectively (P < 0.05).

### 3.5 Effects of exogenous SA on the content of osmotic adjustment substances in *Leymus chinensis* seedlings under extreme drought

**Figure 7:**
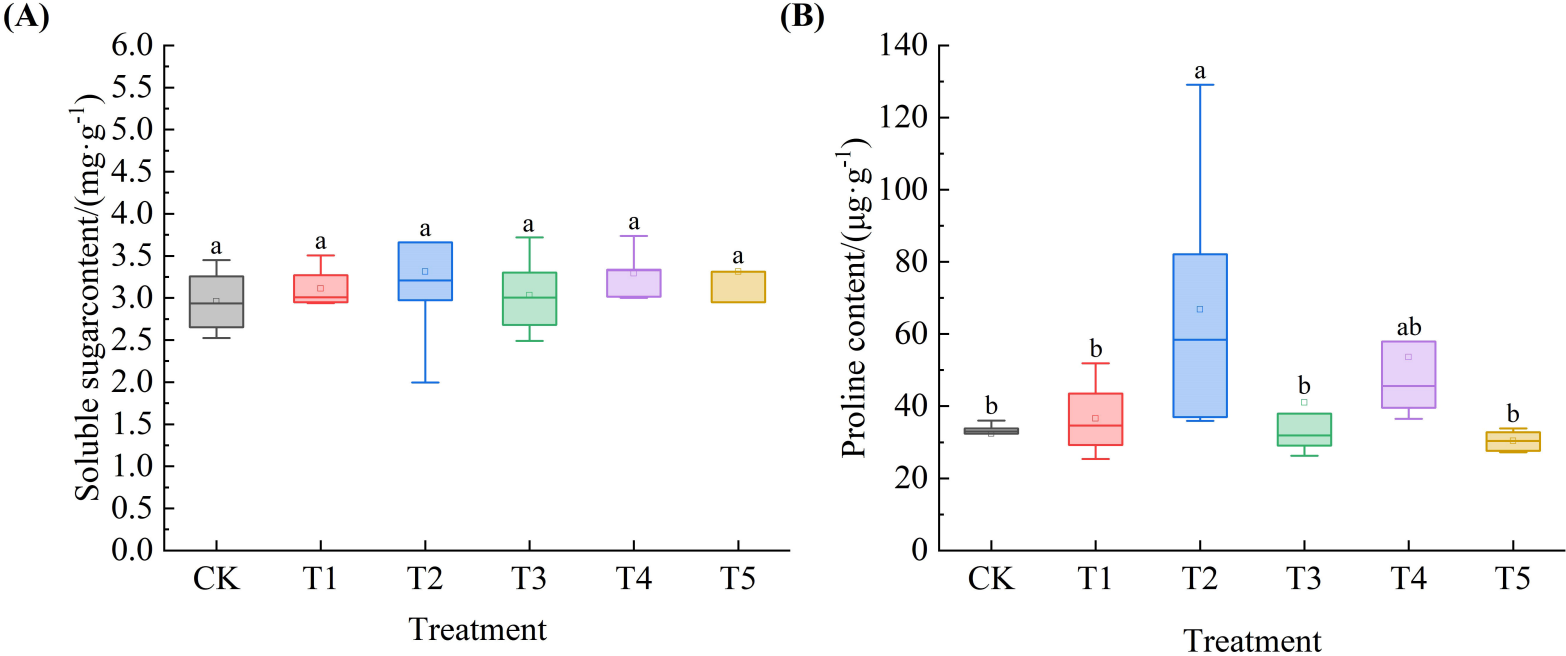
Effects of exogenous salicylic acid (SA) on the contents of osmotic adjustment substances in *Leymus chinensis* seedlings under extreme drought conditions. **(A)** Soluble sugar content (SS), **(B)** Proline content (Pro). Different lowercase letters indicate statistically significant differences at *p* < 0.05.

As shown in **Figure 7A**, the SS content in *Leymus chinensis* seedlings increased to varying degrees with increasing SA concentration compared to the control (CK), by 5.2%, 11.9%, 2.5%, 11.3%, and 11.9%, respectively. In **Figure 7B**, the Pro content exhibited a trend of first increasing and then decreasing as SA concentration increased. Notably, under T2 treatment, Pro content increased by 106.4% relative to CK, showing a significant difference (P < 0.05).

### 3.6 Effects of exogenous SA on the oxidative stress response system of *Leymus chinensis* seedlings under extreme drought

**Figure 8:**
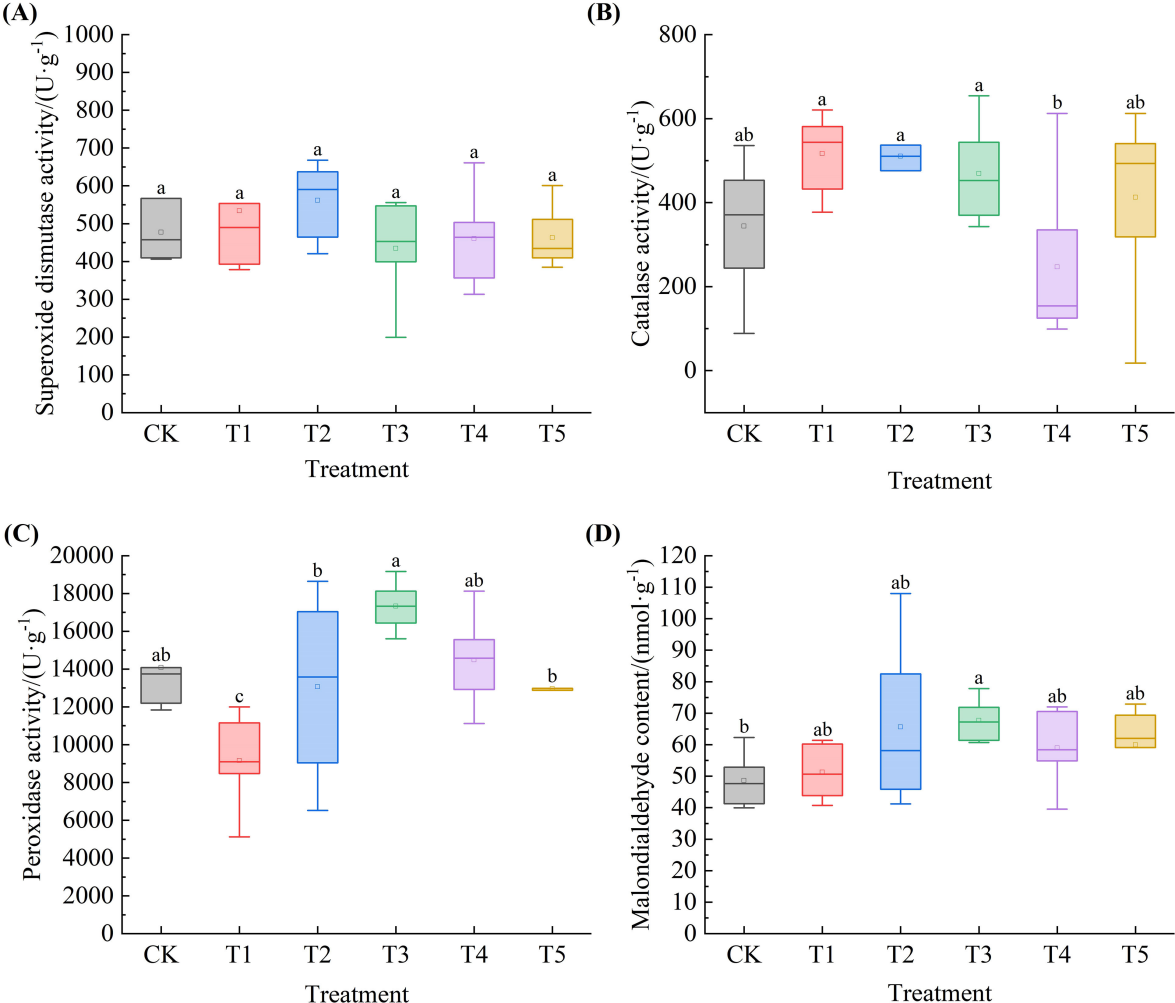
Effects of exogenous salicylic acid (SA) on antioxidant enzyme activities and malondialdehyde (MDA) content in *Leymus chinensis* seedlings under extreme drought conditions. **(A)** Superoxide dismutase (SOD) activity, **(B)** Catalase (CAT) activity, **(C)** Peroxidase (POD) activity, **(D)** Malondialdehyde (MDA) content. Different lowercase letters indicate statistically significant differences at *p* < 0.05.

Under extreme drought conditions, as SA concentration increased, the contents of SOD, POD, and MDA in *Leymus chinensis* seedlings exhibited an initial increase followed by a decrease, whereas CAT content showed an initial decrease followed by an increase. As shown in **Figure 8**, compared with the control (CK), SOD content increased by 11.8% and 17.7% under T1 and T2 treatments, respectively. CAT content increased by 50.1%, 48.3%, 36.4%, and 19.9% under T1, T2, T3, and T5, respectively, relative to CK. POD content decreased by 34.9%, 7.2%, and 7.9% under T1, T2, and T5, with a significant reduction observed under T1 (P < 0.05). MDA content increased by 5.4%, 35.0%, 39.3%, 21.3%, and 23.3% across the five SA treatments compared to CK, with a significant elevation under T3 (P < 0.05).

### 3.7 Comprehensive evaluation of five SA concentrations based on principal component analysis

The comprehensive performance of *Leymus chinensis* under different concentrations of SA was evaluated using principal component analysis (PCA). Seventeen physiological and biochemical indicators of *Leymus chinensis* were summarized into three principal component axes. The analysis revealed six principal components with eigenvalues greater than 1 across SA treatments. Their contribution rates were 23.453%, 14.248%, 10.284%, 10.186%, 8.525%, and 7.994%, respectively, cumulatively accounting for 74.690% of the total variance in the comprehensive performance of *Leymus chinensis* under extreme drought. This cumulative variance exceeds the commonly accepted threshold of 70%, indicating sufficient reliability for evaluating overall plant performance. The eigenvector for each indicator was obtained by dividing its loading value by the square root of the corresponding principal component’s eigenvalue. By multiplying the standardized factor scores (X) by the eigenvectors (Chen et al., 2024), where X_1_–X_17_ represent the standardized data for RWC, Chl, A, g_s_, E, WUE, ΦPSII, F_0_, F_m_, qP, NPQ, SS, Pro, MDA, SOD, CAT, and POD, respectively, the comprehensive score equation for each principal component was derived as follows:

Substituting the standardized data into the equation:


$$y=0.23y_{1}+0.14y_{2}+0.10y_{3}+0.10y_{4}+0.0.9y_{5}+0.08y_{6}$$

The comprehensive evaluation scores for the six SA treatments were calculated. A higher y value indicates better drought resistance performance, with the ranking order as T2 > T3 > T4 > T1 > T5 > CK. According to **Tables 1** and **2**, the comprehensive performance scores of the control group (CK) and the T1 (0.25 mmol/L) and T5 (4.00 mmol/L) treatments were all negative, indicating that under extreme drought conditions, the growth and development of *Leymus chinensis* were significantly impaired, and both excessively low and high SA concentrations compromised the effectiveness of the drought resistance enhancement. The highest comprehensive score was observed under T2 (0.50 mmol/L) treatment, followed by T3 and T4. These results suggest that application of 0.50, 1.00, and 2.00 mmol/L SA solutions can effectively improve the drought tolerance of *Leymus chinensis* under extreme drought stress, with 0.50 mmol/L exhibiting the most pronounced effect and thus significantly enhancing the plant’s adaptability to adverse climatic conditions.

**Table 1:** Formula for the coefficient matrix of the comprehensive performance score of *Leymus chinensis* under extreme drought with different concentrations of SA applied.

The coefficient matrix formula of the comprehensive performance score of *Leymus chinensis* under extreme drought conditions with different concentrations of SA applied.
y_1_ = 0.364X_1_-0.364X_2_+0.359X_3_+0.346X_4_+0.313X_5_+0.286X_6_+0.284X_7_+0.271X_8_-0.204X_9_+0.025X_10_-0.277X_11_-0.045X_12_+0.118X_13_-0.093X_14_+0.074X_15_+0.020X_16_-0.050X_17_
y_2_=-0.033X_1_-0.003X_2_+0.266X_3_+0.270X_4_-0.052X_5_-0.144X_6_+0.137X_7_+0.084X_8_+0.531X_9_+0.458X_10_+0.407X_11_-0.168X_12_-0.086X_13_-0.108X_14_+0.275X_15_+0.144X_16_+0.070X_17_
y_3_ = 0.166X_1_-0.078X_2_+0.129X_3_+0.166X_4_-0.307X_5_+0.126X_6_-0.158X_7_-0.187X_8_+0.084X_9_-0.137X_10_+0.260X_11_+0.474X_12_+0.456X_13_-0.185X_14_+0.144X_15_-0.409X_16_-0.059X_17_
y_4_=-0.113X_1_+0.192X_2_-0.075X_3_+0.012X_4_+0.383X_5_+0.391X_6_-0.202X_7_+0.009X_8_+0.146X_9_+0.347X_10_-0.022X_11_+0.136X_12_+0.347X_13_+0.533X_14_-0.035X_15_-0.032X_16_+0.194X_17_
y_5_ = 0.249X_1_+0.359X_2_-0.233X_3_-0.193X_4_-0.020X_5_+0.197X_6_+0.122X_7_+0.257X_8_+0.001X_9_-0.063X_10_+0.023X_11_+0.357X_12_-0.019X_13_-0.102X_14_+0.459X_15_+0.455X_16_-0.193X_17_
y_6_=-0.244X_1_-0.063X_2_+0.253X_3_+0.140X_4_-0.207X_5_-0.036X_6_-0.112X_7_+0.138X_8_-0.057X_9_-0.347X_10_-0.139X_11_+0.153X_12_-0.120X_13_+0.203X_14_+0.322X_15_+0.162X_16_+0.652X_17_

**Table 2:** Ranking of comprehensive performance scores for *Leymus chinensis* under extreme drought stress across different exogenous SA concentrations.

Treatment	y_1_	y_2_	y_3_	y_4_	y_5_	y_6_	y	Sorting
CK	-2.35	-0.21	0.00	-0.02	0.22	-0.66	-0.61	6
T1	-0.21	0.13	1.18	-0.03	-1.51	0.45	-0.02	4
T2	2.20	-0.58	0.00	0.87	0.08	0.60	0.57	1
T3	0.56	1.46	-0.58	-0.50	1.03	0.39	0.35	2
T4	0.88	-0.36	-0.48	-0.01	0.28	-0.95	0.05	3
T5	-1.07	-0.44	-0.12	-0.32	-0.10	0.17	-0.35	5

### 3.8 Structural equation model analysis of the growth characteristics of *Leymus chinensis* under extreme drought treated with exogenous SA

**Figure 9:**
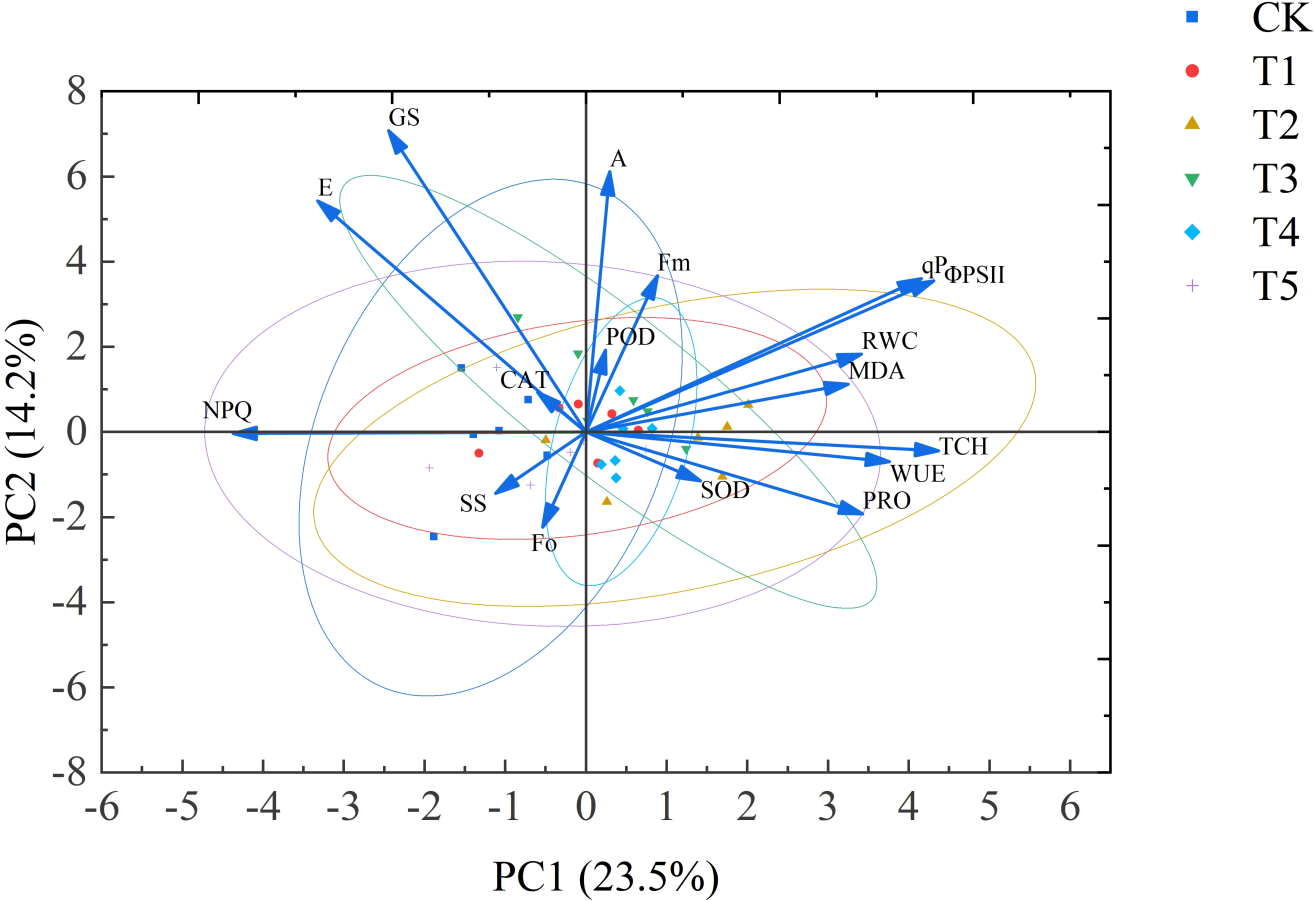
Principal component analysis of physiological and biochemical indices in *Leymus chinensis* seedlings.

**Figure 10:**
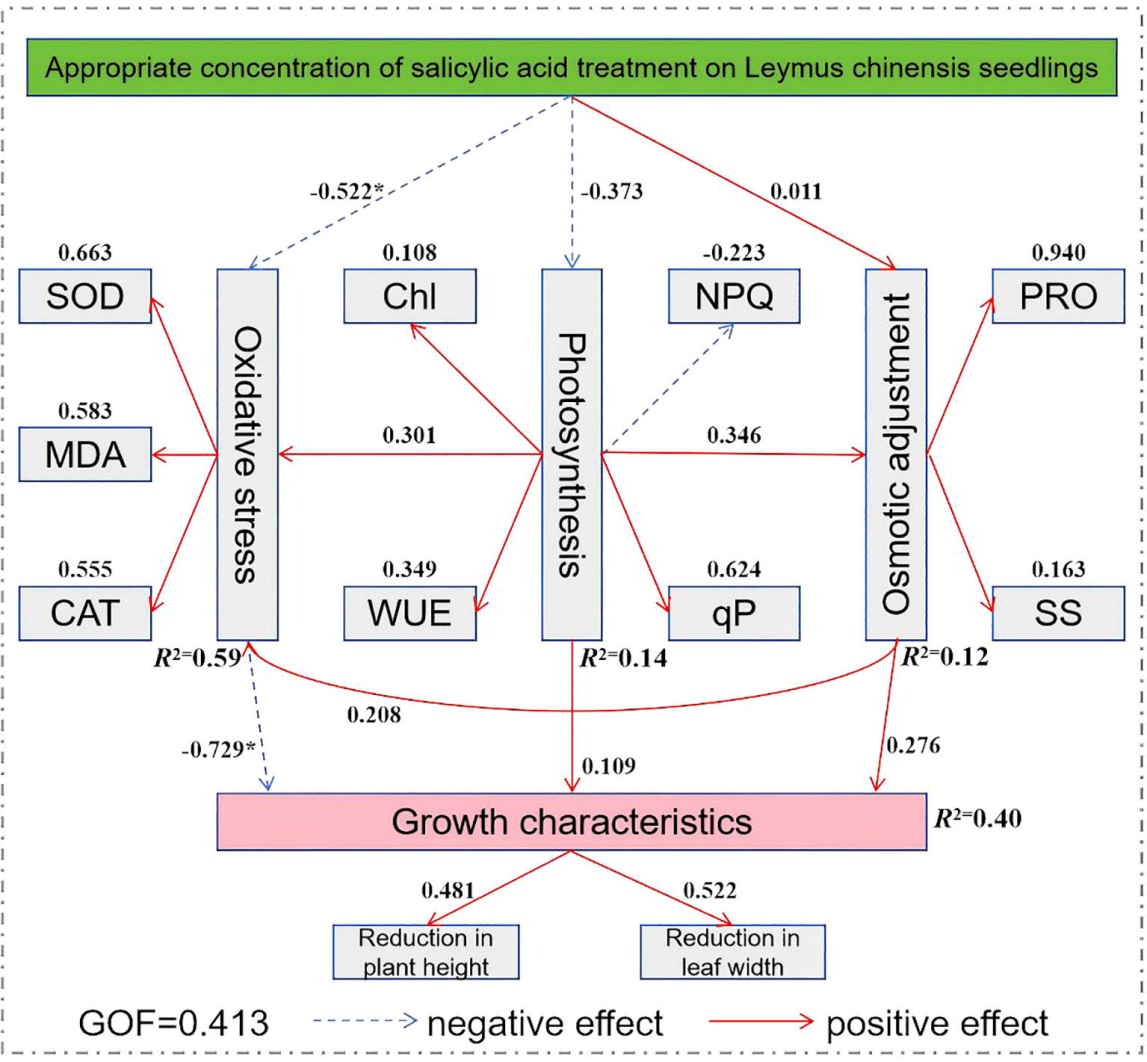
Structural equation model of exogenous salicylic acid (SA) regulating the growth and morphological structure of *Leymus chinensis*. Red solid arrows indicate positive effects, and blue dashed arrows indicate negative effects (*P < 0.05). GOF represents the goodness of fit of the structural equation model, and *R*^2^ represents the percentage of variance of the dependent variable explained by the model.

In **Figure 9**, 14 observed variables that showed significant contributions to principal components 1 and 2, along with 5 latent variables and 18 samples (see **Table 3**), were selected to construct the model. Subsequently, the plspm package was used in RStudio to fit the model and generate the path diagram. As shown in **Figure 10**, in the process of exogenous SA regulating the growth characteristics of *Leymus chinensis* seedlings under extreme drought, the path coefficients from the oxidative stress system to growth characteristics were -0.522 and -0.729, respectively, and both were statistically significant. The model reveals that the path coefficient of appropriate SA concentration on the oxidative stress system is significantly negative. Although the principal component comprehensive scores for concentrations ranging from 0.5 to 2.0 mmol/L are all positive, their effects differ in magnitude. The structural equation model shows a stronger preference for 0.5 mmol/L, which aligns with the previously observed “low-concentration promotion and high-concentration inhibition” pattern. Within the oxidative stress module, SOD, CAT, and MDA exhibit positive contribution values of 0.663, 0.555, and 0.583, respectively, indicating that increased levels of these components positively influence the oxidative stress system in the model. Thus, the higher MDA content in SA-treated groups compared to the control (CK) may be attributed to SA enhancing the tolerance threshold of *Leymus chinensis* seedlings to MDA accumulation, while the clearance capacity of the antioxidant system has not yet fully compensated, leading to transiently elevated MDA levels. In the photosynthesis module, the path coefficient of SA on photosynthetic performance is -0.373, potentially influenced by gs and Tr; however, the nonsignificant coefficient suggests this pathway is not a dominant regulatory mechanism. In the osmotic adjustment module, the path coefficient of SA is 0.011, indicating a slight positive effect on osmolyte accumulation, but its statistical insignificance precludes it from being considered a core mechanism. Finally, the path coefficient from the oxidative stress system to growth characteristics is -0.729 and statistically significant, demonstrating that activation of the oxidative stress response mitigates the decline in plant height and leaf width, thereby prolonging drought resistance in *Leymus chinensis* seedlings.

**Table 3:** Construction of the measurement model for exogenous SA treatment at appropriate concentrations.

Latent variables	Observation variables
SA concentration	0.50mmol/L
	1.00mmol/L
	2.00mmol/L
Photosynthesis	Chlorophyll (Chl)
	Water use efficiency (WUE)
	Non-photochemical quenching coefficient (NPQ)
	Photochemical quenching coefficient (qP)
Osmotic adjustment	Proline content (Pro)
	Soluble sugar (SS)
Oxidative stress	Superoxide dismutase activity (SOD)
	Catalase activity (CAT)
	Malondialdehyde (MDA)
Growth form	The reduction in plant height
	The reduction range of leaf width

## 4 Discussion

### 4.1 Effects of SA on physiological and biochemical indicators of *Leymus chinensis* seedlings

Under drought stress, plant cell division is inhibited, leading to suppressed growth and reduced biomass (Cui et al., 2024; Zhang et al., 2024). The results indicate that although all treatments reduced plant height, the decline was minimal under T3, suggesting a superior capacity for growth maintenance under water deficit conditions. This enhanced growth resilience may be attributed to the significant improvement in RWC. Leaf relative water content serves as an indirect indicator of water deficit and cellular vitality in plants under drought conditions (Sapes and Sala, 2021). The relative water content (RWC) of *Leymus chinensis* seedlings under all five treatments was higher than that of the control (CK), with the T3 treatment achieving the highest value. This demonstrates that SA mitigates leaf water loss under extreme drought stress, with optimal efficacy observed at the T3 concentration—consistent with findings from studies on corn and sesame (Shao et al., 2018; Pourghasemian et al., 2020). The enhanced RWC is closely associated with the accumulation of osmotic adjustment substances. Pro content increased significantly under T2, while SS content rose progressively across all SA treatments. These results indicate that SA effectively promotes osmotic adjustment, enabling cells to maintain turgor pressure and sustain metabolic activity, thereby supporting improved growth maintenance—aligning with previous reports on SA application in cotton (Das et al., 2024).

Chlorophyll is a green pigment in plants, and its content level can reflect the intensity of photosynthesis to a certain extent (Li et al., 2018). This study demonstrates that appropriate SA application helps maintain relatively high Chl levels in *Leymus chinensis* seedlings under extreme drought stress, thereby enhancing their tolerance to drought-induced adverse effects. This observation aligns with findings from SA studies in Scrophularia ningpoensis (Shohani and Fazeli, 2025). This study further revealed that an appropriate concentration of SA treatment induced a decoupling between Pn and gs in Leymus chinensis seedlings: at T4, Pn was enhanced while gs and Tr were consistently reduced—a pattern highly consistent with previous findings in Arabidopsis thaliana (Hirosawa et al., 2021). This decoupling suggests that the promotion of photosynthesis by SA is independent of stomatal regulation, a conclusion supported by structural equation modeling and further substantiated by the significant improvement in WUE observed at T4. Nevertheless, this beneficial effect exhibits a distinct concentration threshold. Beyond the T4 concentration, the progressive decline in gs becomes a limiting factor for photosynthesis, ultimately resulting in reduced Pn, thereby demonstrating the characteristic concentration-dependent duality of SA in modulating photosynthetic performance (Dianat et al., 2016).

Drought stress typically leads to increased Fo and decreased Fm (Lotfi et al., 2024), as it damages the PSII reaction center, resulting in enhanced fluorescence leakage (Zhang et al., 2019). The reduction in Fo under T2 and T3 treatments indicates alleviated damage to the PSII reaction centers, while the significant increase in Fm under T3 suggests enhanced structural stability of these centers—consistent with findings from SA studies in wheat (Abdi et al., 2022). Furthermore, the most compelling evidence comes from ΦPSII and quenching parameters. In all SA-treated plants, ΦPSII was significantly elevated, particularly at T1 and T2. This improvement is likely attributable to a shift in energy utilization strategy: SA-treated seedlings exhibited higher qP and lower NPQ, indicating that SA promotes a more efficient allocation of absorbed light energy, directing a greater proportion toward photochemical reactions rather than thermal dissipation. This enhancement of primary photochemistry directly accounts for the observed increase in Pn, independent of stomatal regulation. Chlorophyll fluorescence parameters thus provide an internal physiological perspective that supports and explains the gas exchange data, revealing the core mechanism by which SA improves photosynthetic performance under drought stress.

Plant resistance to drought stress is closely associated with the activity of endogenous antioxidant enzymes. Previous studies have demonstrated that under severe drought conditions, antioxidant enzyme activities decline significantly (Liu et al., 2025). In this study, extreme drought was found to reduce the activities of SOD, CAT, and POD in Leymus chinensis seedlings, whereas treatment with an appropriate concentration of salicylic acid (SA) maintained these enzymes at relatively high levels. These findings suggest that SA may enhance antioxidant enzyme activity to scavenge drought-induced superoxide radicals, mitigate membrane lipid peroxidation, and thereby preserve membrane integrity and sustain normal cellular physiological metabolism (Zamani et al., 2024).

Under drought stress, intensified membrane lipid peroxidation leads to the accumulation of malondialdehyde (MDA), which can trigger protein and nucleic acid denaturation, reduce membrane fluidity, and increase membrane permeability (Yin et al., 2024). Consequently, MDA content is widely regarded as a key indicator of plant injury under abiotic stress (Seleiman et al., 2021). Notably, in this study, SA-treated seedlings did not exhibit lower MDA levels compared to the control (CK); instead, MDA content was slightly elevated. We propose two mechanistic explanations for this observation, grounded in the dynamic nature of oxidative stress and plant stress tolerance: First, oxidative stress is inherently dynamic (Qin et al., 2022). Under extreme drought, the initial rate of lipid peroxidation may surpass the scavenging capacity of the SA-induced antioxidant system, resulting in rapid early accumulation of MDA (Yadav et al., 2020). Although SA enhances antioxidant enzyme activity, its primary role may be in suppressing ongoing oxidative damage rather than eliminating pre-existing MDA, which requires longer-term metabolic turnover—thus explaining the higher MDA levels detected at the sampling time point (Wang et al., 2022). Second, SA may elevate the tolerance threshold of the antioxidant defense system in Leymus chinensis by stabilizing membrane structure or activating repair pathways, enabling cells to maintain physiological functionality even under elevated MDA concentrations. This implies that survival under stress does not solely depend on minimizing MDA but may involve adaptive resilience mechanisms (Yang et al., 2023). Collectively, the increased MDA content reflects the cumulative burden of lipid peroxidation under extreme drought, indicating that while SA does not fully prevent membrane damage, it effectively attenuates its progression, highlighting its protective role as a mitigator rather than a complete blocker of oxidative injury.

### 4.2 Mechanism of exogenous SA in enhancing drought resistance of *Leymus chinensis* seedlings

Integrated analysis of physiological, biochemical indices and structural equation modeling reveals that SA enhances drought resistance in *Leymus chinensis* seedlings primarily through coordinated modulation across three key dimensions: water relations, photosynthetic function optimization, and oxidative stress management. With respect to water relations, SA-induced moderate reduction in gs effectively minimizes transpirational water loss, while simultaneously promoting the accumulation of osmotic adjustment substances, thereby enhancing cellular water retention by lowering osmotic potential. The synergistic action of these mechanisms enables sustained turgor pressure maintenance, as evidenced by the significant increase in RWC. In terms of photosynthetic performance, moderate SA concentrations maintain or slightly elevate Pn despite general gs limitation, indicating that SA’s non-stomatal regulatory effects predominate over stomatal regulation. Chlorophyll fluorescence analysis further supports this: enhanced stability of PSII reaction centers, significantly increased ΦPSII and qP, coupled with a sharp decline in NPQ, demonstrate that SA redirects absorbed light energy toward photochemical utilization rather than thermal dissipation. This strategic reallocation of excitation energy constitutes the core intrinsic mechanism enabling plants to overcome stomatal constraints and sustain carbon assimilation under drought. At the oxidative stress level, SA operates via a “management” rather than complete suppression strategy. SA application at optimal concentrations activates key antioxidant enzymes—SOD and CAT—thereby enhancing reactive oxygen species (ROS) scavenging capacity. However, the lack of corresponding reduction in MDA levels indicates that under extreme drought, ROS generation may transiently exceed the scavenging capacity of the induced antioxidant system, resulting in accumulated lipid peroxidation damage. Thus, SA’s beneficial role likely lies in attenuating the rate of oxidative injury and, potentially through membrane stabilization or repair activation, increasing cellular tolerance to oxidative stress. This adaptive strategy does not prevent damage entirely but mitigates its progression and buys critical time for survival and post-stress recovery.

### 4.3 Cost analysis and feasibility evaluation of exogenous SA in practical agricultural applications

#### 4.3.1 Cost analysis and feasibility evaluation of exogenous SA in practical agricultural applications

During the experiment, 30 ml of SA solution was applied to each pot of *Leymus chinensis*, with an effective concentration range of 0.5–2.00 mmol/L. Accordingly, the required amount of analytical grade SA per pot ranged from 65.0545 g to 260.2180 g. Based on this dosage, field application would require 942 L of SA solution per hectare per treatment, resulting in a cost of 9.76 to 39.03 yuan per hectare.

#### 4.3.2 Feasibility assessment of exogenous SA application in practical agricultural settings

From an economic perspective, the maximum cost of SA application per hectare is only 39.03 yuan, indicating high cost-effectiveness. Operationally, the procedure is straightforward and involves minimal steps: after weighing the required amount of SA, it is simply dissolved in water and sprayed. The process is clear, easy to implement, and has a high tolerance for operational variability. Positive effects are consistently observed within the recommended concentration range, making it accessible and effective for farmers with varying levels of education and technical experience. Therefore, exogenous SA application demonstrates considerable feasibility for practical agricultural production.

## 5 Conclusion

The application of exogenous SA effectively mitigates the adverse effects of drought stress on *Leymus chinensis* seedlings. The efficacy of SA is strongly concentration-dependent, with beneficial effects observed at low to moderate concentrations and inhibitory effects at higher levels. Among the five tested concentrations, 0.50, 1.00, and 2.00 mmol/L exhibited favorable outcomes, with 0.50 mmol/L yielding the optimal response. The mechanism of SA action is multifaceted, involving improved water status, enhanced photosynthetic performance, and coordinated regulation of osmotic adjustment substances and antioxidant systems. These findings from optimal concentration screening and mechanistic analysis provide robust evidence for the potential use of SA in alleviating abiotic stress and offer novel insights into enhancing drought resistance in *Leymus chinensis*.

## Data Availability

The original contributions presented in the study are included in the article/supplementary material. Further inquiries can be directed to the corresponding author.
